# Inoculation With the Plant-Growth-Promoting Rhizobacterium *Pseudomonas fluorescens* LBUM677 Impacts the Rhizosphere Microbiome of Three Oilseed Crops

**DOI:** 10.3389/fmicb.2020.569366

**Published:** 2020-10-09

**Authors:** Jesús A. Jiménez, Amy Novinscak, Martin Filion

**Affiliations:** ^1^Biology Department, Université de Moncton, Moncton, NB, Canada; ^2^Agriculture and Agri-Food Canada, Saint-Jean-sur-Richelieu Research and Development Center, Saint-Jean-sur-Richelieu, QC, Canada

**Keywords:** *Pseudomonas fluorescens*, *Brassica napus*, *Glycine max*, *Buglossoides arvensis*, rhizosphere, microbiome

## Abstract

The bacterial communities inhabiting the rhizosphere play an important role in plant development and health. Here we studied the effect of inoculation with *Pseudomonas fluorescens* LBUM677, a plant growth promoting rhizobacterium that promotes seed oil accumulation, on the rhizosphere microbiome of three oilseed crops (*Brassica napus*, *Buglossoides arvensis*, and *Glycine max*) over time. Next-Generation high-throughput sequencing targeting the V4 region of 16S rDNA was used to characterize the microbial communities associated with the three different crops, inoculated or not with LBUM677, over a time period of up to 90 days post-inoculation. A total of 1,627,231 amplicon sequence variants were obtained and were taxonomically grouped into 39 different phyla. LBUM677 inoculation and sampling date were found to significantly influence the rhizosphere microbiome of the three oil-producing crops under study. Specifically, inoculation with LBUM677 and sampling date, but not the plant species, were found to significantly alter the alpha- and the beta-diversity of the rhizosphere microbial communities. Differential abundance analyses found that 29 taxonomical bacterial groups were significantly more abundant in the LBUM677 treatments while 30 were significantly more abundant in the control treatments. Predicted functions of the microorganisms were also enriched, including 47 enzymatic pathways in LBUM677 treatments. These non-targeted effects on rhizosphere bacterial communities are discussed in the context of oilseed crops.

## Introduction

The soil region surrounding plant roots, known as the rhizosphere, is a highly complex matrix supporting rich microbial communities, and is considered as one of the most dynamic interfaces on Earth (Berendsen et al., 2012; Del Carmen Orozco-Mosqueda et al., 2018). Microbial interactions occurring in the rhizosphere are of crucial importance for plant health and productivity (Schmeisser et al., 2007; Philippot et al., 2013). In order to promote beneficial microbial interactions in the rhizosphere, there is an increasing interest in inoculating plants with beneficial microorganisms (Aeron et al., 2011). Among these organisms, plant growth promoting rhizobacteria (PGPR) represent a functional group including diverse bacterial taxa capable of promoting plant nutrient availability, growth, and disease suppression (Kloepper et al., 1980; Chabot et al., 1993; Edwards et al., 2015; Morrison et al., 2017). Among PGPRs, *Pseudomonas* spp. represent a group of significant importance that has been widely studied in agriculture (Pathma et al., 2011; Santoro et al., 2015; Kong et al., 2016).

The microbiome of the plant, comprising all microorganisms associated to a plant’s rhizosphere, phyllosphere, and endosphere play an important part in the plant’s health and growth and has even been referred to as the plant’s second genome (Berendsen et al., 2012; Compant et al., 2019). The plant roots directly influence the rhizosphere through the secretion of a variety of compounds, including organic acids, amino acids, fatty acids, sugars and vitamins creating a nutrient-rich location for microbial growth (Del Carmen Orozco-Mosqueda et al., 2018; Compant et al., 2019; Pascale et al., 2020; Saad et al., 2020). The rhizosphere was also found to contain a greater microbial species richness than the endosphere or the phyllosphere (Rodriguez et al., 2019). Many studies have demonstrated that the plant’s genotype and edaphic characteristics like soil nutrient concentration, pH and moisture content, may have a significant impact on rhizosphere microbial communities (Lemanceau et al., 1995; Bakker et al., 2015). It is also of interest to investigate and document the impact that PGPR inoculations have on indigenous soil microbiomes as this is a growing research area (Martínez-Hidalgo et al., 2019). Previous studies that have measured the non-target effects of PGPR on the indigenous bacterial community have demonstrated contrasting results. Many studies found that a significant effect is generally rare, transient, and spatially limited. Some examples include inoculation of nitrogen-fixing *Azospirillum* strains in tomato (*Solanum lycopersicum* L.), wheat (*Triticum sativum* L.), and maize (*Zea mays* L.) rhizospheres (Bashan et al., 1995; Castro-Sowinski et al., 2007; Felici et al., 2008) and various *Pseudomonas* strains in maize, barley (*Hordeum vulgare* L.), and potato (*Solanum tuberosum* L.) rhizospheres (Castro-Sowinski et al., 2007; Buddrus-Schiemann et al., 2010; Roquigny et al., 2018). However, there have also been studies that have shown a direct effect on the plant microbiome due to bacterial inoculations. Examples include inoculations of *Rhizobium* species in faba beans (*Vicia faba* L.) (Zhang et al., 2010) and soybean (*Glycine max* L.) (Zhang et al., 2011), *Proteus vulgaris* in Kimchi cabbage (*Brassica rapa* L.) (Bhattacharyya and Lee, 2016), and *Pseudomonas* species in maize (Kozdrój et al., 2004; Kozdrój, 2008). The advent of metagenomic sequencing to examine the effects of microbial inoculation on the non-target soil microbiome will be a research area that will see great developments in the near future (Martínez-Hidalgo et al., 2019). In this context, non-target effects of inoculation with selected *Pseudomonas* spp. on the rhizosphere microbiome of oilseed crops has, to our knowledge, never been studied before.

We have previously demonstrated that *P. fluorescens* strain LBUM677 (hereafter LBUM677) can significantly promote lipid accumulation in three different oilseed crops (Jiménez et al., 2020): canola (*Brassica napus* L.) and soybean (*Glycine max* L.), two crops with a considerable seed oil content and great commercial importance (Ruddle et al., 2013), as well as corn gromwell (*Buglossoides arvensis* L.), an oilseed crop with an increasing nutraceutical interest due to its unusual seed accumulation of the omega-3 stearidonic acid (Cumberford and Hebard, 2015). In the present study, we wanted to determine the effect of LBUM677’s inoculation over time on the indigenous rhizosphere bacterial communities associated with these three different oilseed crops.

## Materials and Methods

### Bacterial Inoculum and Seeds

*P. fluorescens* LBUM677 (Genbank accession CP038438.1) was originally isolated from the rhizosphere of strawberry plants cultivated in Bouctouche, NB, Canada. LBUM677 was grown in Tryptic Soy Broth (Becton, Dickinson and Company, Burlington, Canada), incubated at 25°C with agitation at 200 rpm for 48 h and the bacterial concentration was adjusted to 1 × 10^9^ cells mL^–1^ based on OD_600 nm_ measurements and a previously determined growth curve. *G. max* and *B. napus* seeds were obtained from Pioneer Hi-Bred (Mississauga, ON, Canada) and *B. arvensis* seeds were obtained from Technology Crops International (Kensington, PE, Canada).

### Growth Chamber Experiment, Rhizosphere Soil Sampling, and DNA Extraction

The experimental set-up consisted of three plant species (*G. max*, *B. napus*, and *B. arvensis*) inoculated with LBUM677 or water (negative control), sampled at three time points (30, 60, and 90 days post-inoculation) using four replicates per treatment and time combination for each plant species. A complete randomized block design was used for a total of 72 experimental units. The experiments were conducted in a PGR15 growth chamber (Conviron, Winnipeg, MB, Canada) under the following conditions: 20°C, 80% relative humidity and a 16 h photoperiod at 500 μmol (m^2^)^–1^s^–1^. The soil used was obtained from the Senator Hervé J. Michaud Agriculture and Agri-Food Canada Research Farm (Bouctouche, NB, Canada) and was characterized as a Gleyed Podzolic Gray Luvisol (GLPZ.GL), per the Canadian Soil Classification System with 62% sand, 25% silt, 13% of clay, 2.6% organic matter and a pH of 5.2 (Canada, 1987). Seeds of *G. max*, *B. napus*, or *B. arvensis* were sown 1 cm deep in pots containing 400 g of soil. Ten milliliter of either LBUM677 inoculum (1 × 10^9^ cells mL^–1^) or water (for control treatments) was added to the seeds at sowing. The pots were initially watered 24 h after the inoculation and then every 2 days. Fertilization was carried out 30 d after sowing and then every 2 weeks using 100 mL of Hoagland solution per pot (Hoagland and Arnon, 1938). Rhizosphere soil sampling (destructive sampling) was carried out at 30, 60, and 90 days post-inoculation by shaking the plants to remove loosely adhering soil and collecting the soil remaining on the roots. Rhizosphere soil was immediately frozen in liquid nitrogen to prevent nucleic acid degradation and then lyophilized using a lyophilizer (Thermo Fisher Scientific, Mississauga, ON, Canada). Samples were stored at −80°C until DNA extraction. DNA was extracted from 0.25 g of rhizosphere soil using the method described in Griffiths et al. (2008). DNA quantity was measured using the Quanti-It PicoGreen dsDNA Assay Kit (Molecular Probes, Eugene, OR, United States).

### PCR Reaction, Illumina MiSeq Sequencing, and Sequence Analysis

PCR amplification of the V4 region of the bacterial 16 rRNA gene and Illumina sequencing was performed by the Genome Quebec and McGill University Genome Centre (Montreal, QC, Canada). The 515F and 806R primers were used to target the V4 region and were designed to be universal for bacterial and archaeal taxa (Bates et al., 2011; Bergmann et al., 2012). Purified amplicons were pooled in equimolar concentrations and paired-end sequenced (2 × 250) on an Illumina MiSeq platform.

The raw paired-end reads were processed using the QIIME2 pipeline (v2019.10) (Bolyen et al., 2019). Briefly, raw FASTQ files were demultiplexed and quality filtered using the q2-demux plugin followed by denoising with the DADA2 pipeline (Callahan et al., 2016) to identify amplicon sequences variants (ASVs) (Callahan et al., 2017). All ASVs were aligned with mafft (Katoh, 2002) and used to construct a phylogenetic tree with fasttree2 (Price et al., 2010). Alpha-diversity metric (Shannon’s diversity index), beta-diversity metric (weighted UniFrac) (Lozupone and Knight, 2005), and Principle Coordinate Analysis (PCoA) were estimated following rarefaction of samples to 6,000 sequences per samples. A rarefaction depth of 6,000 sequences was determined to retain the most samples and to assure adequate sequencing depth of each sample (three samples were removed from subsequent analyses due to having less than 6,000 sequences). Taxonomy was assigned to ASVs using a naïve Bayes taxonomy classifier against the Silva 138 99% OTUs reference sequences (Quast et al., 2013). Metagenome functions were predicted using the PICRUSt2 pipeline in QIIME2 (Langille et al., 2013).

### Statistical Analyses

Statistical analyses were performed using the QIIME2 pipeline (Bolyen et al., 2019). Kruskal-Wallis tests were used to determine statistical significance of Shannon’s diversity index between treatments, sampling dates, or plant species. Permutational multivariant analysis of variance (PERMANOVA) was used to determine statistically significant differences of the weighted UniFrac beta-diversity measurement between treatments, sampling dates, or plant species. Differences in bacterial abundance and the enzyme pathways between treatments were calculated using linear discriminant analysis (LDA) effect size (LEfSe) (Segata et al., 2011).

All sequences generated in this study have been deposited in DDBJ/EMBL/GenBank under the BioProject ID number: PRJNA634202.

## Results

### Sequencing Results and Diversity Metrics Effects

**FIGURE 1:**
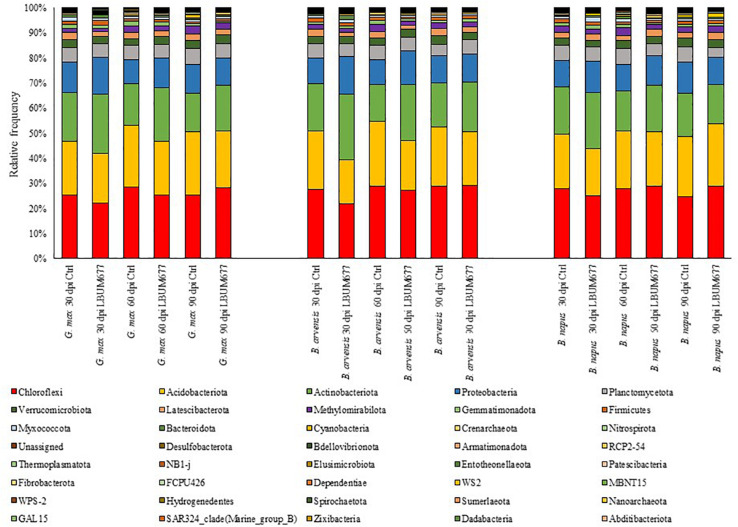
Bar plot of the relative abundance of the bacterial taxa at the class level for each plant, date, and treatment combination under study.

During the course of the experiment three plants died, and their rhizosphere soil was not sampled (sample *B. arvensis* 60 days Control rep 4; sample *B. napus* 60 days LBUM677 rep 3; and sample *B. napus* 90 days LBUM677 rep 4). Following the Illumina sequencing, a total of 3,904,076 raw sequences were obtained and following the DADA2 filtering, these sequences were clustered into 1,627,231 ASVs. Each library (one library per sample) contained between 1,810 and 46,980 ASVs, with an average of 23,583 (Supplementary Table S1). All samples were subsequently rarefied to 6,000 ASVs for alpha- and beta-diversity analyses and to confirm that Illumina sequencing depth was adequate (Supplementary Figure S1). Three samples were removed from the subsequent analyses because they contained less than 6,000 ASVs (sample *B. arvensis* 30 days Control rep 3; sample *B. arvensis* 60 days Control rep 3; and *B. napus* 60 days Control rep 4). Taxonomy was assigned to the ASVs and they were found to be associated to 39 different phyla (Figure 1). Sequences that could not be classified into any known group were assigned to the group Unassigned. The most dominant phyla across the samples were *Chloroflexi, Acidobacteria, Actinobacteria*, and *Proteobacteria*, the sum of which comprised more than 79% of the total reads in every library, representing on average 27, 22, 19, and 12% of all ASVs, respectively (Figure 1). 21 phyla were detected in all libraries, while only two phyla were found to be exclusively associated with the rhizosphere of one plant species, namely *B. napus* (phyla Dadabacteria and Abditibacteriota), although these phyla comprised very small percentages of the total reads (less than 1% in both cases).

**FIGURE 2:**
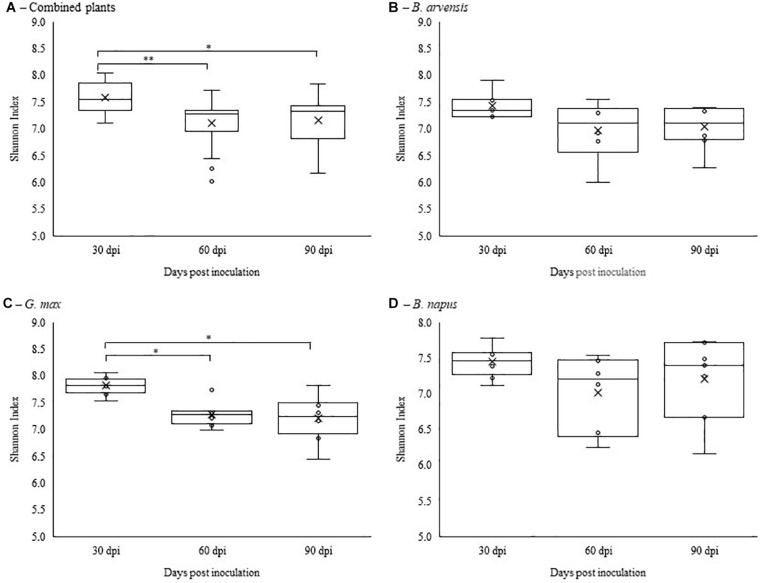
Temporal variations of alpha-diversity metrics in the bacterial microbiome of three oil-producing crops. **(A)** Alpha-diversity metric (Shannon’s index) of temporal variations for all plants and treatments combined. **(B)** Alpha-diversity metric (Shannon’s index) of temporal variations for *B. arvensis* plants and treatments combined. **(C)** Alpha-diversity metric (Shannon’s index) of temporal variations for *G. max* plants and treatments combined. **(D)** Alpha-diversity metric (Shannon’s index) of temporal variations for *B. napus* plants and treatments combined. Kruskal-Wallis pair-wise test was used to assess statistical significance between groups (**p < 0.001, *p < 0.05).

**FIGURE 3:**
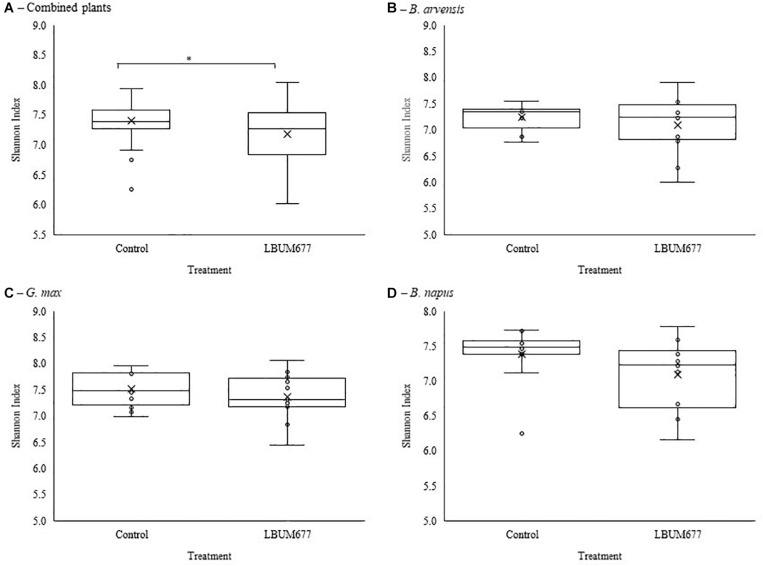
Treatment variations of alpha-diversity metrics in the bacterial microbiome of three oil-producing crops. **(A)** Alpha-diversity metric (Shannon’s index) of treatment variations for all plants and sampling dates combined. **(B)** Alpha-diversity metric (Shannon’s index) of treatment variations for *B. arvensis* plants and sampling dates combined. **(C)** Alpha-diversity metric (Shannon’s index) of treatment variations for *G. max* plants and sampling dates combined. **(D)** Alpha-diversity metric (Shannon’s index) of treatment variations for *B. napus* plants and sampling dates combined. Kruskal-Wallis pair-wise test was used to assess statistical significance between groups (*p < 0.05).

**FIGURE 4:**
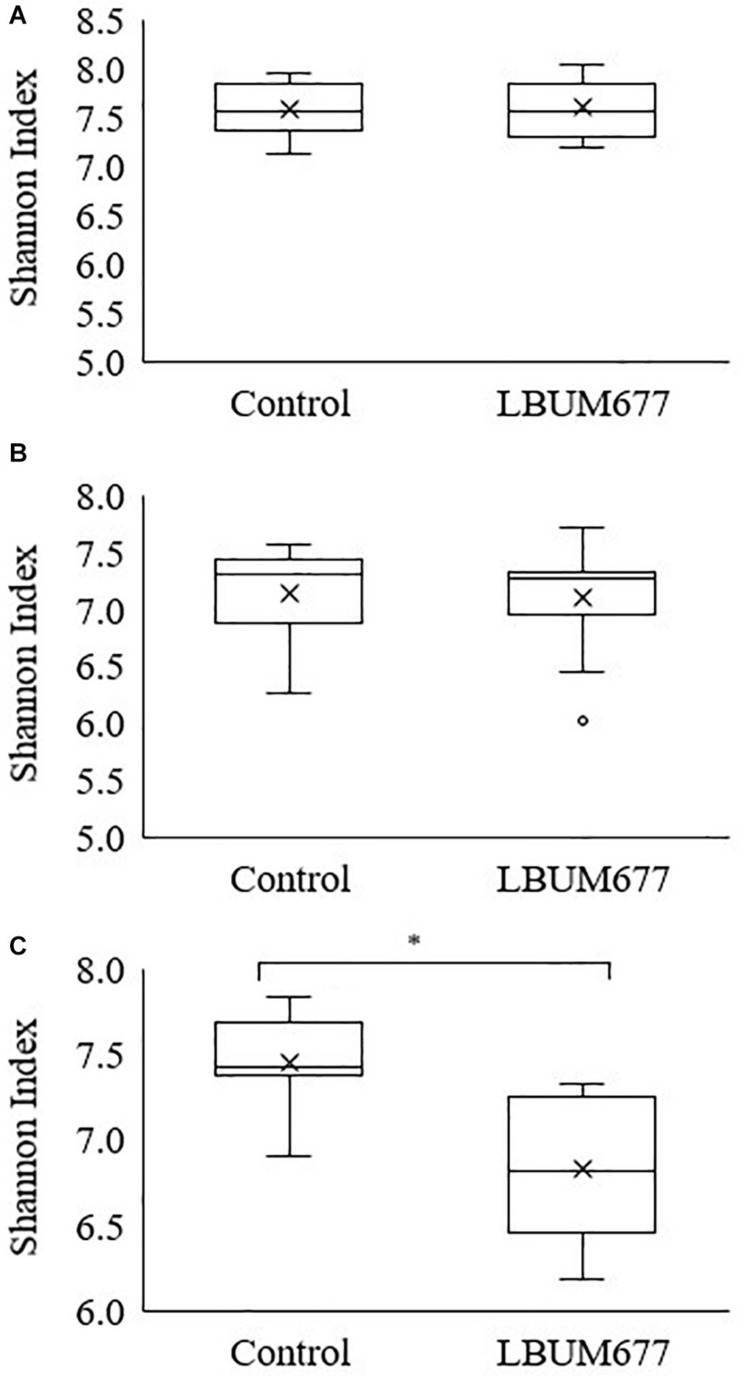
Temporal and treatment variations of alpha-diversity metric in the bacterial microbiome of three oil-producing crops. Alpha-diversity metric (Shannon’s index) of temporal variations amongst treatments at **(A)** 30 days, **(B)** 60 days, and **(C)** 90 days post-inoculation. Kruskal-Wallis pair-wise test was used to assess statistical significance between treatments (*p < 0.05).

Alpha diversity was calculated using Shannon’s diversity index. An initial general analysis was performed by grouping the results for all plant species together. A second analysis was performed where the alpha diversity was calculated for each plant species separately. For all analyses, significant differences in the diversity of the bacterial communities were measured by Kruskall-Wallis tests. For the general analysis, differences in alpha diversity was observed when comparing the three sampling dates (Figure 2A) and when comparing the two treatments: inoculated with LBUM677 and control (Figure 3A). Specifically, for the sampling date analysis, a significant difference was found between 30 days post-inoculation (dpi) and 60 dpi (*H* = 13.16; *p* = 0.0009) and between 30 and 90 dpi (*H* = 11.22; *p* = 0.001; Figure 2A). No difference in the diversity of bacterial communities was found between 60 and 90 dpi (*H* = 0.26; *p* = 0.6), indicating that the bacterial diversity was greatest at 30 days and decreased thereafter. Treatment with LBUM677 was found to significantly decrease the diversity of the rhizosphere samples (*H* = 4.0; *p* = 0.04) (Figure 3A). Additionally, no general effect of plant species was observed (Supplementary Figure S2A). The general effect of sampling date on the microbiome was further decomposed by LBUM677 treatment for each time-point. This analysis showed that the only significant difference between the LBUM677-treated and control microbiomes was observed at 90 dpi (*H* = 12.74; *p* = 0.0004) (Figure 4).

When examining the effects of sampling date on each plant species independently, it was found that the sampling date had a significant effect only on the bacterial communities found in the rhizosphere of *G. max* (Figure 2C). Specifically, a significant difference was found between 30 and 60 dpi (*H* = 9.93; *p* = 0.005) and between 30 and 90 dpi (*H* = 8.65; *p* = 0.005), while no significant effects were found in the bacterial communities of *B. arvensis* (Figure 2B) or *B. napus* (Figure 2D). When examining the effects of the bacterial treatment, it was found that there was no significant difference in the alpha diversity of both groups for each plant species (Figures 3B–D).

**FIGURE 5:**
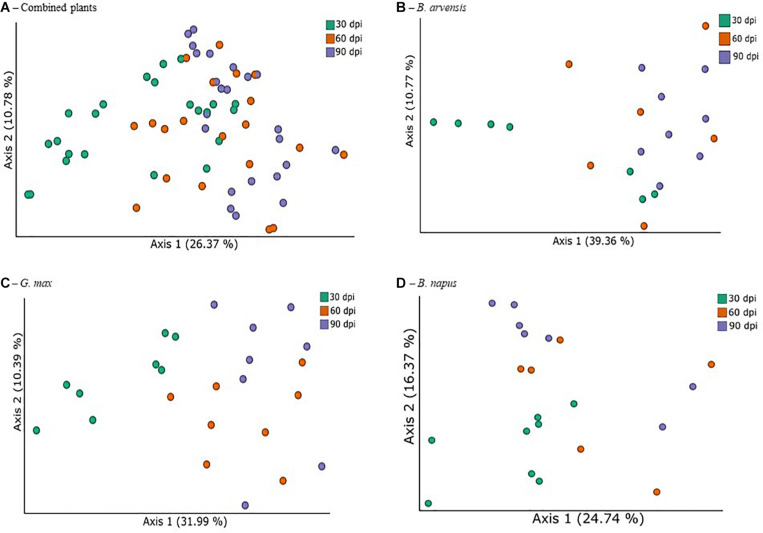
Temporal variations of beta-diversity metrics in the bacterial microbiome of three oil-producing crops. **(A)** PCoA plot representative of temporal beta-diversity variations using weighted UniFrac metric for all plants and treatments combined (*F* = 5.51, *p* = 0.001). Each point represents a sample, **(B)** PCoA plot representative of temporal beta-diversity variations using weighted UniFrac metric for all *B. arvensis* plants and treatments combined (*F* = 2.67, *p* = 0.005). Each point represents a sample, **(C)** PCoA plot representative of temporal beta-diversity variations using weighted UniFrac metric for all *G. max* plants and treatments combined (*F* = 4.01, *p* = 0.001). Each point represents a sample, **(D)** PCoA plot representative of temporal beta-diversity variations using weighted UniFrac metric for all *B. napus* plants and treatments combined (*F* = 2.19, *p* = 0.002). Each point represents a sample.

**FIGURE 6:**
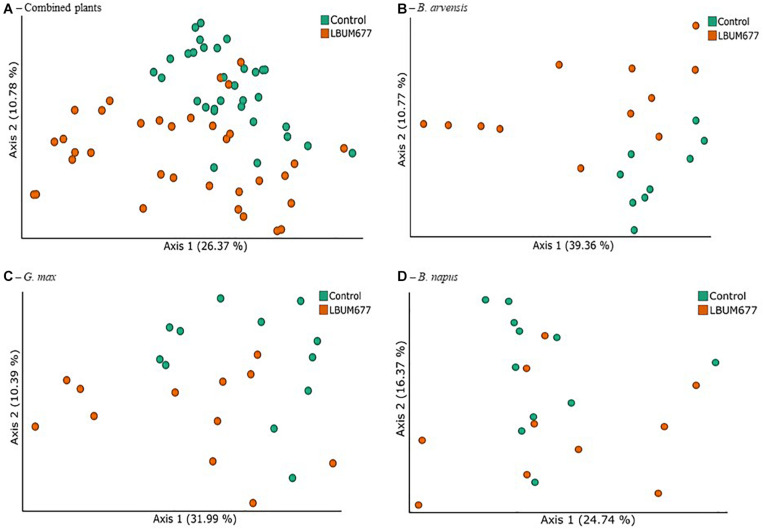
Treatment variations of beta-diversity metrics in the bacterial microbiome of three oil-producing crops. **(A)** PCoA plot representative of treatment beta-diversity variations using weighted UniFrac metric for all plants and sampling dates combined (*F* = 5.51, *p* = 0.001). Each point represents a sample, **(B)** PCoA plot representative of treatment beta-diversity variations using weighted UniFrac metric for all *B. arvensis* plants and sampling dates combined (*F* = 3.20, *p* = 0.011). Each point represents a sample, **(C)** PCoA plot representative of treatment beta-diversity variations using weighted UniFrac metric for all *G. max* plants and sampling dates combined (*F* = 2.41, *p* = 0.021). Each point represents a sample, and **(D)** PCoA plot representative of treatment beta-diversity variations using weighted UniFrac metric for all *B. napus* plants and sampling dates combined (*F* = 1.52, *p* = 0.106). Each point represents a sample.

In order to identify the treatments associated with compositional dissimilarity in microbiota, we examined the beta-diversity between samples using the weighted UniFrac measure. Initial tests were performed by grouping the results for all plant species together (Figure 5A). Three tests were performed using PERMANOVA to analyze either the effect of sampling time, plant species, or treatment, irrespective of the other two effects. Statistically significant differences in the beta-diversity were only found when comparing the effect of sampling date or of LBUM677 treatment (*F* = 5.51; *p* = 0.001). For the sampling date analysis, a significant difference was found between the 30 and 60 dpi samples (*F* = 5.13; *p* = 0.001), between 30 and 90 dpi (*F* = 8.25; *p* = 0.001) and 60 and 90 dpi (*F* = 1.62; *p* = 0.04). Finally, the effect of plant species on the bacterial community was found to be not significant (Supplementary Figure S2B). Principal coordinates analysis based on weighted UniFrac distances between samples showed differences between sampling time (Figure 5A) and LBUM677 inoculation (Figure 6A). The first two principal components explained 37.15% of the total variation.

Following the general analysis, the effect of sampling time and bacterial treatment was examined using the weighted UniFrac beta-diversity measure. For *B. arvensis*, statistically significant differences were found when comparing the sampling dates (*F* = 2.67; *p* = 0.005) and treatments (*F* = 3.20; *p* = 0.011). For *G. max*, statistically significant differences were found when comparing the sampling dates (*F* = 4.01; *p* = 0.001) and treatments (*F* = 2.41; *p* = 0.021). Finally, for *B. napus*, statistically significant differences were found only when comparing the sampling dates (*F* = 2.19; *p* = 0.002) and not the treatments (*F* = 1.52; *p* = 0.106). Principal coordinates analysis based on weighted UniFrac distances between samples showed differences between sampling time and LBUM677 inoculation for *B. arvensis* (Figures 5B, 6B), *G. max* (Figures 5C, 6C), and *B. napus* (Figures 5D, 6D).

### Differentially Abundant Bacteria Across Samples

**FIGURE 7:**
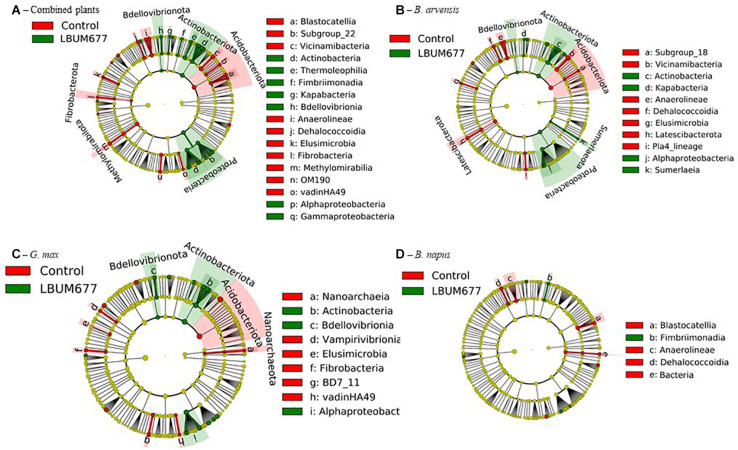
Cladograms representing the differentially abundant taxa between control samples and LBUM677-inoculated samples. **(A)** the combination of all three plant species, **(B)** *B. arvensis* plants, **(C)** *G. max* plants and **(D)** *B. napus* plants. Red indicates an increased abundance in control samples while green indicates an increase in LBUM677-treated samples. Differentially abundant sequences were determined using the non-parametric factorial Kruskal-Wallis sum-rank test. A subsequent Linear Discriminant Analysis was used to estimate the effect size of each differentially abundant feature.

**FIGURE 8:**
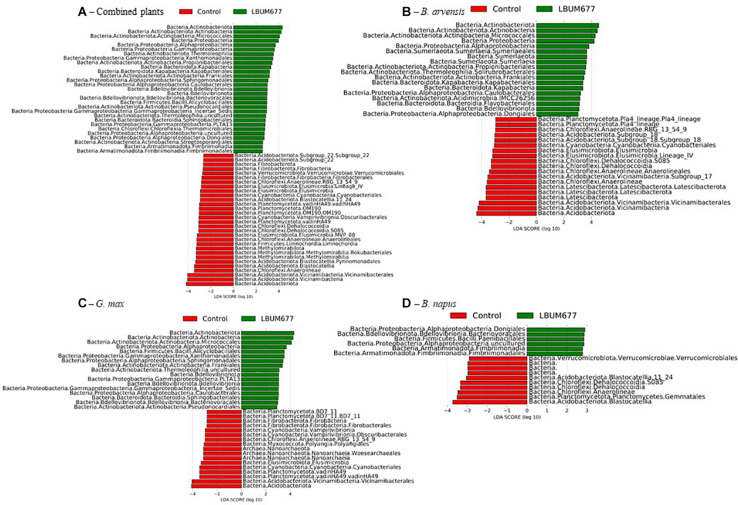
Histogram of the LDA scores computed for differentially abundant taxa between control samples and LBUM677-treated samples as determined using LEfSe. **(A)** the combination of all three plant species, **(B)** *B. arvensis* plants, **(C)** *G. max* plants, and **(D)** *B. napus* plants. Red indicates an increased abundance in control samples while green indicates an increase in LBUM677-treated samples. Differentially abundant sequences were determined using the non-parametric factorial Kruskal-Wallis sum-rank test. A subsequent Linear Discriminant Analysis was used to estimate the effect size of each differentially abundant feature.

To further examine the effects of LBUM677 inoculation on the rhizosphere microbiome, we investigated which taxa were differentially abundant between the two groups using LeFSE. A general analysis was performed with all plant species combined followed by analyses of each plant species individually. For the general analysis, Twenty-nine bacterial groups were found to be more abundant in the rhizosphere of plants treated with LBUM677, while 30 bacterial groups were found to be more abundant in the rhizosphere of control plants (Figures 7A, 8A). Taxonomic classification of the differentially abundant groups to the order level was attempted but was not possible in all cases. In the LBUM677 treatments, *Actinobacteria* (nine groups), *Alpha-Proteobacteria* (five groups), *Gamma-Proteobacteria* (four groups), *Bdellovibrionota* (three groups), *Armatimonadota* (two groups), and *Bacteropodota* (two groups) were the only phyla that showed enrichment with more than one bacterial order, while in the control group, *Acidobacteria* (eight groups), *Chloroflexi* (five groups), *Planctomycetota* (four groups), *Elusimicrobiota* (three groups), *Methylomirabilota* (three groups), *Fibrobacterota* (three groups), and *Cyanobacteria* (two groups) were the only phyla containing more than one enriched order.

When examining which taxonomic groups were differentially abundant in the rhizosphere of each plant species, it was found that *B. arvensis* had 14 differentially abundant taxa in the LBUM677 treatment and 19 in the control, *G. max* had 17 differentially abundant taxa in the LBUM677 and control treatments while *B. napus* had 6 differentially abundant taxa in the LBUM677 treatments and 10 in the controls (Figures 7B–D, 8B–D). Interestingly, when examining the individual groups that were differentially abundant, some phyla were noted as differentially abundant in the *B. arvensis* samples that were not in the general analysis (for example, Sumerlaeota, and Latescibacterota), while in the *G. max* samples, archaea were found to be more abundant in the control samples. Finally, *B. napus* showed the smallest number of differentially abundant taxa in the LBUM677-treated and control samples.

### Predicted Genome Functions

**FIGURE 9:**
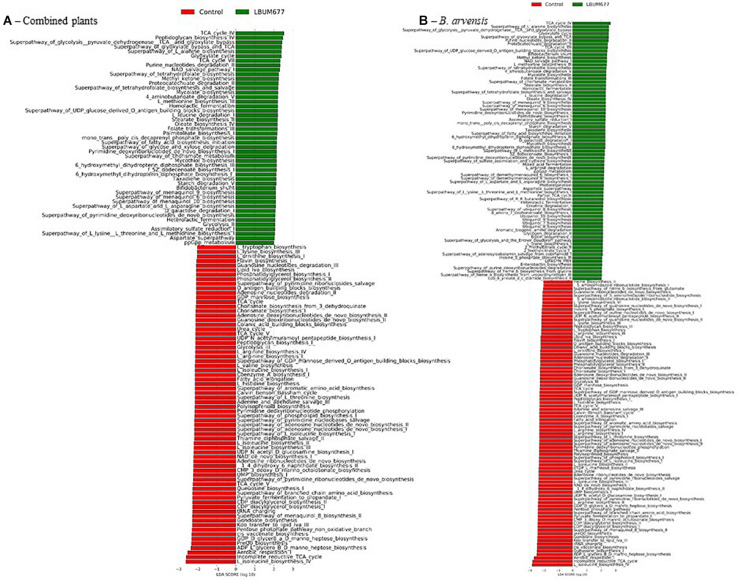
Histogram of the LDA scores computed for differentially abundant MetaCyc pathways in control samples and in LBUM677-treated samples as determined using LEfSe. **(A)** the combination of all three plant species and **(B)** *B. arvensis* plants. Differentially abundant sequences were determined using the non-parametric factorial Kruskal-Wallis sum-rank test. A subsequent Linear Discriminant Analysis was used to estimate the effect size of each differentially abundant feature.

The analytic pipeline Phylogenetic Investigation of Communities by Reconstruction of Unobserved States (PICRUSt) (Langille et al., 2013) was used to predict functions based on bacterial taxa. The PICRUSt results gave abundances of MetaCyc pathways which were subsequently analyzed in the LeFSE pipeline. A general analysis, combining all plant species found that 47 pathways were significantly more abundant in the LBUM677-treated rhizospheres while 70 pathways were more abundant in the control rhizospheres (Figure 9A). Some of the more abundant pathways in the LBUM677 treatment includes pathways involved in the tricarboxylic acid cycle (TCA), including TCA cycle IV, cycle V, cycle VI, and cycle VII and certain menaquinol biosynthesis pathways, including those for menaquinol-6, menaquinol-9, and menaquinol-10. Certain pathways more abundant in the control treatments include the super-pathways of various cellular compounds, including pyrimidine, L-isoleucine, L-lysine, and L-tryptophan.

**FIGURE 10:**
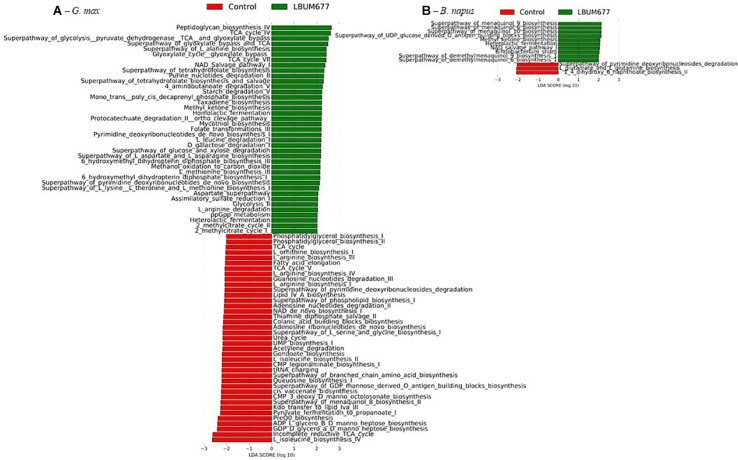
Histogram of the LDA scores computed for differentially abundant MetaCyc pathways in control samples and in LBUM677-treated samples as determined using LEfSe. **(A)** *G. max* plants and **(B)** *B. napus* plants. Differentially abundant sequences were determined using the non-parametric factorial Kruskal-Wallis sum-rank test. A subsequent Linear Discriminant Analysis was used to estimate the effect size of each differentially abundant feature.

When examining the more abundant pathways by individual plant species, it was observed that 75 pathways were more abundant in the LBUM677-treated rhizosphere of *B. arvensis* plants, while 83 pathways were more abundant in the control samples (Figure 9B). The *G. max* samples were found to have 39 pathways that were more abundant in the LBUM677-treated samples and 39 that were more abundant in the control samples (Figure 10A) while the *B. napus* samples had 10 pathways more abundant in the LBUM677-treated samples and three in the control samples (Figure 10B). The LBUM677-treated samples displayed various pathways that were more abundant, with some including the biosynthesis of ubiquinol-7, -8, -9, and -10 in *B. arvensis*, the superpathway of menaquinol-6, -9, and -10 and the superpathway of demethylmenaquinol-6, and -9 in *B. arvensis* and *B. napus*. The implications of these metabolic functions remain to be explored as these results are based on taxonomical predictions and have not been validated experimentally.

## Discussion

A PGPR inoculation is often deemed successful if the inoculant is found to be beneficial to the plant, either through increased plant biomass accumulation, productivity, or reduction of disease symptoms. A previous study examining the effect of LBUM677 on *B. napus, B. arvensis*, and *G. max* has shown that this bacterium is a successful PGPR inoculant. Specifically, LBUM677 can significantly increase seed oil yield and fatty acids content of the three different plants (Jiménez et al., 2020). Additionally, LBUM677 demonstrated certain general plant growth promotion properties, including significantly increasing total plant weight in *B. napus* and *B. arvensis*, but not in *G. max*. LBUM677 was also found to persist to a similar level in the rhizosphere of the three plants, even though there was a general decrease in its population levels over time (Jiménez et al., 2020). Following the demonstration that LBUM677 is an effective PGPR capable of significantly increasing seed oil accumulation in these three oilseed crops, it was logical to subsequently examine its possible non-target effects on the indigenous soil microbial populations of these three plant species.

Few studies have evaluated the effect that PGPR inoculation has on the soil microbiome (Ambrosini et al., 2016). This information is important to first determine if any significant adverse effects are observed on the soil microbial communities and secondly to better understand the indirect effect that a targeted PGPR inoculation may have on the plant due to specific changes in its microbiome. An important factor to take into consideration when interpreting the results of such studies, is the experimental and technical approach used to characterize the impact of PGPR on the soil microbiome. So far, studies have often relied on low-resolution methods to examine the effects of bacterial inoculations on the indigenous rhizosphere bacterial populations (Kozdrój et al., 2004; Kozdrój, 2008; Zhang et al., 2010, 2011; Ke et al., 2019). Recent advances in metagenomics analyses have led to increased research on non-target effects of bacterial inoculations (Crecchio et al., 2018; Martínez-Hidalgo et al., 2019) and in this study we took advantage of these recent technical developments coupled to a rigorous experimental design to study the impact of LBUM677 on the rhizosphere soil microbiome.

Surprisingly, in our study, only inoculation with LBUM677 and sampling date, and not the plant species under study, significantly influenced the rhizosphere microbiomes of *B. napus, B. arvensis*, or *G. max*. Previous studies have shown that the first microorganisms forming the microbiome of a plant are obtained through vertical transmission by seeds (Compant et al., 2019). Plants have also been shown to actively recruit members of the bulk soil microbial community by modulating their exudates to recruit a particular set of rhizosphere microbes (Pérez-Jaramillo et al., 2016). In general, it is acknowledged that plant genotypes and/or cultivars have at least some influence on their microbiomes. For example, one study attributed 5–7% of the microbiome variation to different maize genotypes (Peiffer et al., 2013), while another study found a significant effect of barley genotype on the diversity of root-associated bacterial communities (Bulgarelli et al., 2015). Other studies found that the microbiome of genetically modified canola was different than the microbiome of conventional varieties (Dunfield and Germida, 2001, 2003). However, it should be mentioned that similarly to our study, some reports indicated no effect of different plant genotypes on their microbiomes. For example, in one study examining the effect of different soybean genotypes on the bacterial communities in the rhizosphere, no significant influence of the different genotypes on the bacterial communities in either pot or field assays was found (Xu et al., 2009). Another study examining the microbiomes associated with five genotypes of perennial wild mustard plants (*Boechera stricta*) showed no effect of the plant genotype on the microbial communities associated with the roots (Wagner et al., 2016). However, in these studies, different genotypes of a single plant species were compared, while in ours, different plant species were instead compared.

Another factor to consider is that in our study, the first rhizosphere sampling was performed 30 days after the seeds were planted; this period of time could have been too long to observe the initial effect of vertical seed transmission. Under our experimental conditions, the sampling date was found to be a significant factor affecting the microbiome population in the rhizosphere of the plants under study. This effect was clearly more pronounced for *G. max* (Figure 2C). These changes were probably due to changes in nutrient availability and different plant development stages, which have previously been shown to have an impact on bacterial populations in response to plant biomass increases and the secretion of compounds and phytochemicals that are produced at different plant growth stages (Chaparro et al., 2014; Yang et al., 2015). Results have shown that the rhizosphere microbiome of soybean (Xu et al., 2009; Wang et al., 2019) and canola (Farina et al., 2012) changes with the plant’s growth stage. It has also been reported that microbiome changes due to PGPR inoculation are generally smaller than those caused by the impact of plant growth (Kröber et al., 2014). For example, a study examining the effects of inoculating *Bacillus velezensis* NJAU-Z9 on pepper seedlings found that differences in the microbial community of the rhizosphere were mainly due to the growth stage of the plant, followed by the introduction of the bacterium (Zhang et al., 2019). Although root exudates of the plants were not examined in this study, previous reports have demonstrated that the root exudates usually change over the course of the different growth stages of the plants and can lead to variations in the microbial community (Yang and Crowley, 2000; Garbeva et al., 2004; Xu et al., 2009; Zhalnina et al., 2018).

The diversity analysis of the rhizosphere microbiomes under study was found to significantly decrease over time (Figure 2) and LBUM677 inoculation was also found to further decrease this diversity (Figure 3). Contrary to this long lasting effect of LBUM677 on the soil microbiome shown in our study, previous work has often concluded that non-target effects due to bacterial inoculation were only transient or inexistent. For example, inoculation with *Pseudomonas* sp. DSMZ 13134 only showed a transient effect on the rhizosphere microbial community structure associated with barley (*Hordeum vulgare*) in the first 3 weeks post-inoculation (Buddrus-Schiemann et al., 2010). *Pseudomonas stutzeri* A1501 inoculation showed no significant microbial enrichments in the rhizosphere of maize (*Zea mays* L.) (Ke et al., 2019) and inoculation of *Pseudomonas synxantha* LBUM223 (previously *Pseudomonas fluorescens*) did not alter the indigenous bacterial populations in the rhizosphere or the geocaulosphere of potato (*Solanum tuberosum* L.) (Roquigny et al., 2018). No significant differences in richness and diversity between inoculated and non-inoculated rhizosphere microbial populations were also observed in lettuce (*Lactuca sativa*) (Cipriano et al., 2016). However, there are also few studies that have instead demonstrated, as in ours, long-lasting differences between the microbial populations of inoculated and non-inoculated rhizospheres. Pre-inoculation of *Bacillus velezensis* in the soil surrounding pepper (*Capsicum annuum* L.) seedlings was found to increase the rhizosphere bacterial richness and diversity (Zhang et al., 2019), while a shift in the community structure of non-inoculated and inoculated rhizosphere bacterial populations was observed in tomato rhizosphere inoculated with *Pseudomonas fluorescens* pc78 (Kong et al., 2016) and in chamomile (*Chamomilla recutita* L.) rhizosphere inoculated with six different PGPR (Schmidt et al., 2014).

It is generally assumed that soil microbial communities with a greater diversity are less susceptible to changes caused by invading microorganisms (Fließbach et al., 2009). Additionally, the soil microbiome is thought to be resilient to the perturbations caused by inoculating non-indigenous bacteria (Moënne-Loccoz et al., 2001). We delved further into the decreasing microbial diversity of the rhizosphere samples under study and compared the diversity of control and LBUM677-treated samples at each time point. These results indicated that even if the diversity was generally decreasing over time, the diversity between the control and LBUM677 samples was only significant at 90 dpi (Figure 4). The results seem to suggest a confounding effect of sampling time and LBUM677 inoculation on the diversity of the rhizosphere microbiomes. This is in part surprising since a general decrease in the population levels of LBUM677 over time in these conditions was also previously demonstrated (Jiménez et al., 2020). In this context, it would be expected that this reduction in the population levels of LBUM677 would not increasingly impact on the rhizosphere microbiome diversity over time. Apparently, the effect of LBUM677 on shaping the rhizosphere microbiome diversity is not population-level dependent, at least under a range of 3 × 10^6^ to 6 × 10^7^ LBUM677 cells g^–1^.

Interestingly, a decrease in the microbial diversity in the rhizosphere of canola and soybean plants during the growth of the plants has also previously been observed. A study examining the effects of the growth stage of canola on the microbial populations in the rhizosphere found that the bacterial richness was 20% larger at the rosette stage and decreased over time as the plant grew to maturity (Farina et al., 2012). For soybean, studies have shown that the abundance and diversity of the bacterial communities in the rhizosphere of the seedling stage were higher than those at the plant’s maturity (Xu et al., 2009; Wang et al., 2019). The decrease in diversity and richness in the rhizosphere microbiome as a plant matures seems counter-intuitive to what is observed in most plant species, however, it might be a particularity observed in the microbiome of specific plant species or, possibly, in the case of oilseed crops.

The most dominant bacterial phyla that were found across the samples in this study were *Chloroflexi, Acidobacteria, Actinobacteria*, and *Proteobacteria*. These phyla are dominant rhizosphere inhabitants with *Actinobacteria* and *Proteobacteria* being two of the four most common phyla associated with plants (Bulgarelli et al., 2013; Levy et al., 2018) and *Acidobacteria* being one of the most abundant phyla found in terrestrial ecosystems (Barns et al., 1999). These phyla have also been found to be dominant in other studies examining the rhizosphere microbiome. A study examining the microbiome of canola found that the most abundant genera were *Agrobacterium*, *Burkholderia*, *Enterobacter*, and *Pseudomonas* (Farina et al., 2012). Four of the five most abundant genera in these samples belonged to the *Proteobacteria* phylum. Studies examining the microbiome of soybean found that the most abundant phyla were *Actinobacteria*, *Acidobacteria*, and *Proteobacteria* in one study (Wang et al., 2019) and in another study they found *Bacteroidetes*, *Nitrospirae*, *Firmicutes*, and *Verrucomicrobia* in addition to the previous phyla to be the most abundant (Xu et al., 2009). *Acidobacteria* have previously been demonstrated to play an important role in carbon cycling due to their ability to degrade complex plant parts, including cellulose and lignin, however, their role in the rhizosphere is not well documented (Ward et al., 2009). Our results showed that 12 bacterial groups were more abundant in the rhizosphere when LBUM677 was inoculated. These groups belong to the *Actinobacteria*, *Proteobacteria*, *Bacteroidetes*, *Planctomycetes*, and *Armatimonadetes* phyla. Many species belonging to *Proteobacteria* and *Actinobacteria* exhibit traits associated with plant growth promotion and disease suppression (Mendes et al., 2011; Zhang et al., 2019), while *Bacteroidetes* contain bacterial species that are involved in nitrogen cycling through denitrification (Chaparro et al., 2014). The *Armatimonadetes* phylum is relatively new and was previously identified as candidate phylum OP10 (Hu et al., 2014). Little is known of its function in the rhizosphere or of that of the *Planctomycetes*.

Functional variations based on differences in the microbial communities using predicted metagenomic pathway analysis were identified. Among these, the metabolic pathways involved in the biosynthesis of ubiquinol-7, -8, -9, and -10, menaquinol-6, -9, and -10 and demethylmenaquinol-6, and -9 were found to be enriched in the LBUM677 treatments (Figure 9). Ubiquinols are widespread in the alpha-, beta-, and gamma-proteobacteria (Pelosi et al., 2016) and are redox-active lipids consisting of a conserved aromatic ring and a polyprenyl hydrophobic tail, with the number of isoprenyl units varying among species (Aussel et al., 2014). Ubiquinol-8 and ubiquinol-9 are common forms among bacteria, and ubiquinol-9 is the major quinone of several large families of aerobic Gram-negative bacteria, including the *Pseudomonadaceae* and the *Rhodospirillaceae*, while ubiquinol-10 has been described in many bacterial species belonging to the *Alphaproteobacteria* (Lester and Crane, 1959). While ubiquinol-9 is their major form, *Pseudomonadaceae* are known to produce smaller amounts of other ubiquinols, including ubiquinol-7 and ubiquinol-8 (Matsushita et al., 1980). Menaquinols and demethylmenaquinols are isoprenoid quinones of the naphthalene series, and are constituents of bacterial plasma membranes, where they play important roles in electron transfer and oxidative phosphorylation. Menaquinols are the most widespread respiratory quinones in biological systems (Fujimoto et al., 2012). Although we did not confirm these functional differences experimentally, these differentially abundant predicted pathways are of interest and may serve as a basis for future studies. The differences in the isoprenoid quinones between bacterial species have been previously used as a taxonomic tool (Pelosi et al., 2016).

In conclusion, this study highlighted the significant effect of *P. fluorescens* LBUM677 inoculation on the rhizosphere microbial communities of three oilseed crops, in a plant-independent manner. Further studies will be needed to determine if these changes observed in the rhizosphere microbiomes contribute, at least in part, to the increased seed oil yield and fatty acids content observed when LBUM677 is inoculated in the rhizosphere or if these microbial changes are simply a consequence of LBUM677 inoculation with no significant impact on plant’s lipid accumulation. Despite our current incapacity to clearly answer this question, the results reported in this study contributed to a better understanding of how non-targeted effects of microbial inoculations impact plant-microbe interactions, and will most probably be useful in the development of specific PGPR-inoculation strategies in oilseed crop agroecosystems.

## Data Availability Statement

The datasets presented in this study can be found in online repositories. The names of the repository/repositories and accession number(s) can be found in the article/Supplementary Material.

## Author Contributions

JJ, AN, and MF contributed to the conception and design of the study. JJ was responsible for part of the sampling, preparing samples for microbiome sequencing, and wrote the first draft of the manuscript. AN was responsible for part of the sampling and microbiome analyses. All authors contributed to manuscript revision, read, and approved the submitted version.

## Conflict of Interest

The authors declare that the research was conducted in the absence of any commercial or financial relationships that could be construed as a potential conflict of interest. A patent has been granted on *P. fluorescens* LBUM677 and its use to enhance total lipid and SDA yields in an oilseed crop (US patent 10,165,743).
