# Supplementary figures and images for: Inoculation With the Plant-Growth-Promoting Rhizobacterium Pseudomonas fluorescens LBUM677 Impacts the Rhizosphere Microbiome of Three Oilseed Crops
Source: Front Microbiol. 2020 Oct 9;11:569366. doi: 10.3389/fmicb.2020.569366 (PMC7581686; doi:10.3389/fmicb.2020.569366)

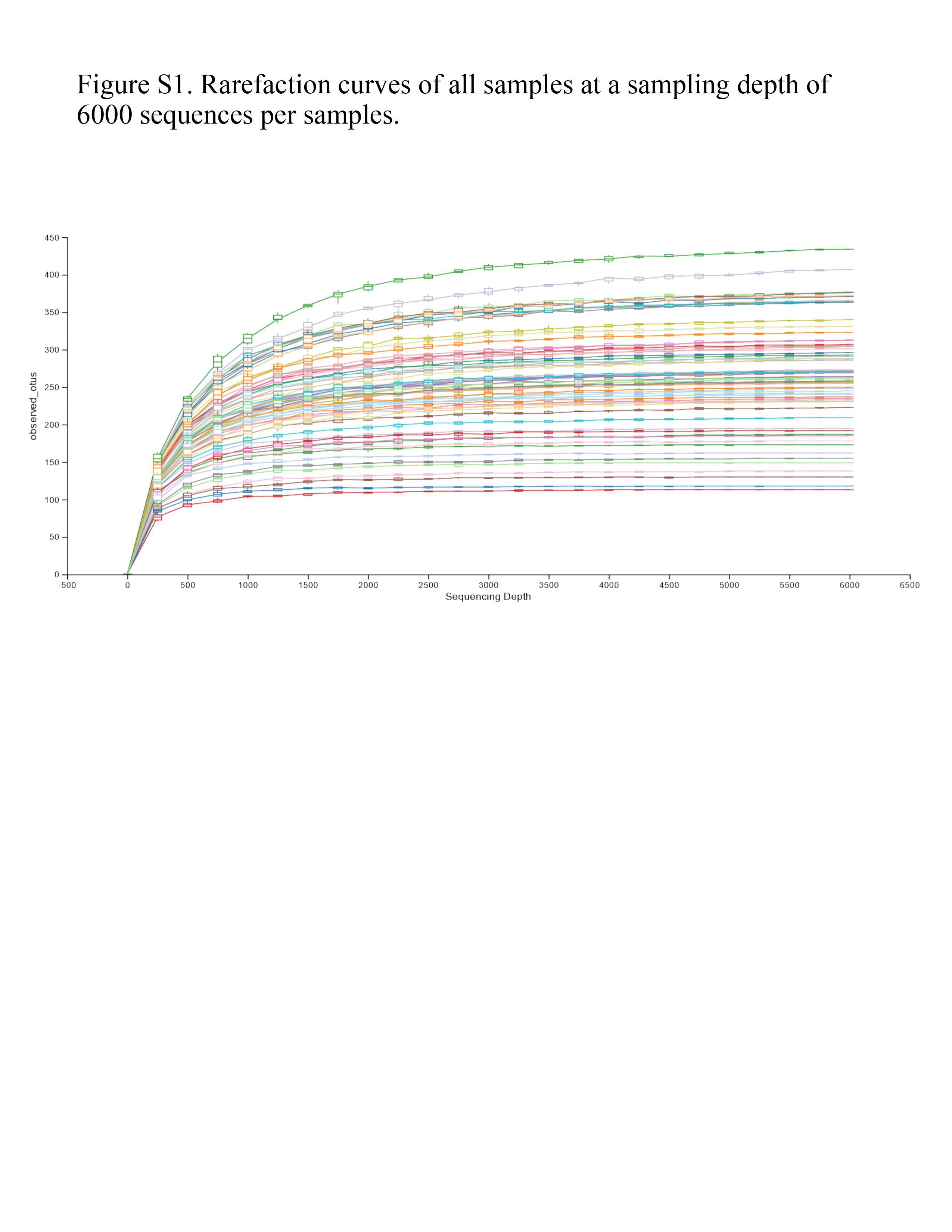

Supplement: Supplementary file 1 [file Image_1.JPEG]

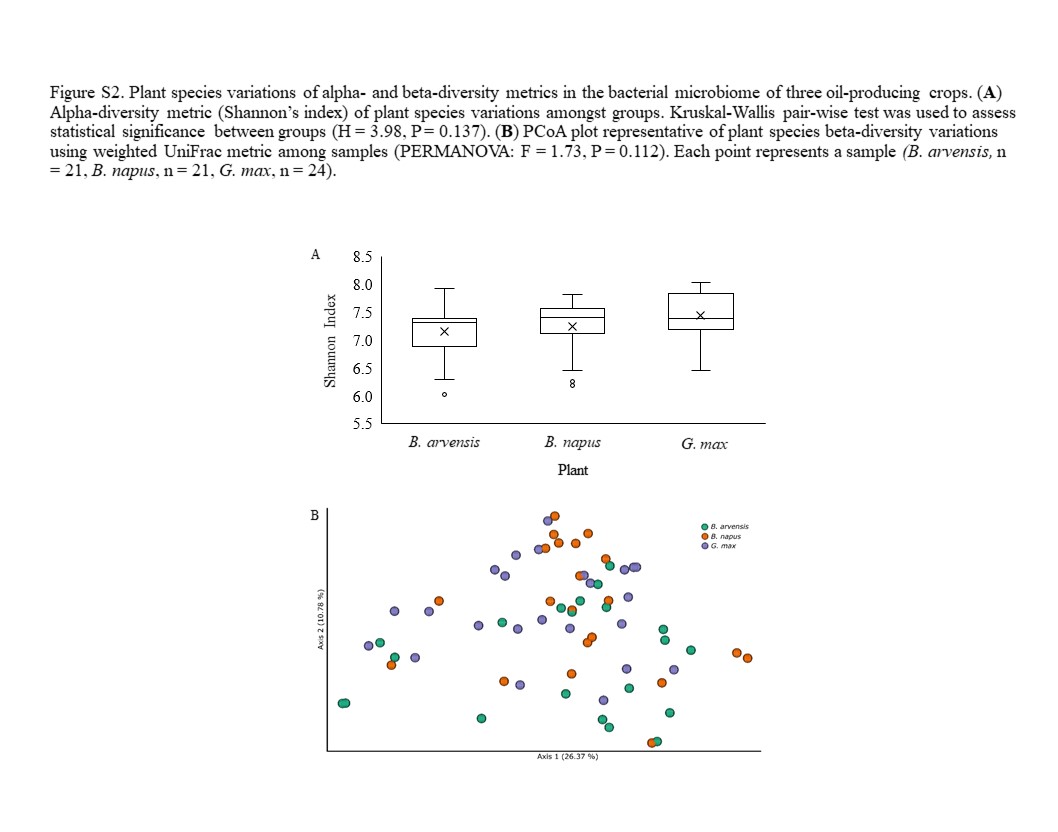

Supplement: Supplementary file 2 [file Image_2.JPEG]
