# Supplementary material for: Inoculation With the Plant-Growth-Promoting Rhizobacterium Pseudomonas fluorescens LBUM677 Impacts the Rhizosphere Microbiome of Three Oilseed Crops
Source: Front Microbiol. 2020 Oct 9;11:569366. doi: 10.3389/fmicb.2020.569366 (PMC7581686; doi:10.3389/fmicb.2020.569366)
Supplement: Supplementary file 3 [file Table_1.docx]

Table S1. MiSeq sequencing results and ASV determination using DADA2 filtering in QIIME2 analysis pipeline

| Sampling date | Treatment | Replicate | Raw sequences | ASVs | |
| --- | --- | --- | --- | --- | --- |
| *Glycine max* | | | | |  |
| 30 days | Control | 1 | 96,714 | 42,883 | |
|  | Control | 2 | 86,407 | 38,613 | |
|  | Control | 3 | 88,712 | 39,733 | |
|  | Control | 4 | 91,540 | 40,196 | |
|  | LBUM677 | 1 | 81,778 | 34,750 | |
|  | LBUM677 | 2 | 76,553 | 33,628 | |
|  | LBUM677 | 3 | 82,536 | 35,569 | |
|  | LBUM677 | 4 | 104,013 | 46,980 | |
| 60 days | Control | 1 | 50,811 | 20,884 | |
|  | Control | 2 | 54,856 | 23,277 | |
|  | Control | 3 | 52,417 | 21,772 | |
|  | Control | 4 | 45,798 | 17,303 | |
|  | LBUM677 | 1 | 58,736 | 23,819 | |
|  | LBUM677 | 2 | 53,612 | 21,975 | |
|  | LBUM677 | 3 | 52,175 | 21,056 | |
|  | LBUM677 | 4 | 87,372 | 38,759 | |
| 90 days | Control | 1 | 58,558 | 25,159 | |
|  | Control | 2 | 80,525 | 35,782 | |
|  | Control | 3 | 73,047 | 30,433 | |
|  | Control | 4 | 46,177 | 19,697 | |
|  | LBUM677 | 1 | 51,019 | 21,284 | |
|  | LBUM677 | 2 | 52,575 | 21,203 | |
|  | LBUM677 | 3 | 41,820 | 16,365 | |
|  | LBUM677 | 4 | 31,035 | 11,232 | |
| *Buglossoides arvensis* | | | | |  |
| 30 days | Control | 1 | 57,698 | 23,055 | |
|  | Control | 2 | 67,720 | 28,780 | |
|  | Control | 3 | 57,537 | 22,571 | |
|  | LBUM677 | 1 | 66,332 | 28,716 | |
|  | LBUM677 | 2 | 58,387 | 24,360 | |
|  | LBUM677 | 3 | 68,061 | 28,812 | |
|  | LBUM677 | 4 | 91,280 | 39,497 | |
| 60 days | Control | 1 | 30,454 | 12,176 | |
|  | Control | 2 | 49,943 | 21,249 | |
|  | LBUM677 | 1 | 19,393 | 6,819 | |
|  | LBUM677 | 2 | 66,665 | 28,320 | |
|  | LBUM677 | 3 | 61,684 | 25,076 | |
|  | LBUM677 | 4 | 36,904 | 14,587 | |
| 90 days | Control | 1 | 36,053 | 14,443 | |
|  | Control | 2 | 44,134 | 18,006 | |
|  | Control | 3 | 56,098 | 24,706 | |
|  | Control | 4 | 62,794 | 24,651 | |
|  | LBUM677 | 1 | 25,101 | 9,554 | |
|  | LBUM677 | 2 | 42,087 | 15,753 | |
|  | LBUM677 | 3 | 45,296 | 17,271 | |
|  | LBUM677 | 4 | 57,717 | 24,214 | |
| *Brassica napus* | | | | |  |
| 30 days | Control | 1 | 73,107 | 30,427 | |
|  | Control | 2 | 73,662 | 31,142 | |
|  | Control | 3 | 59,682 | 25,076 | |
|  | Control | 4 | 50,763 | 19,562 | |
|  | LBUM677 | 1 | 70,231 | 28,426 | |
|  | LBUM677 | 2 | 54,627 | 22,054 | |
|  | LBUM677 | 3 | 63,908 | 27,792 | |
|  | LBUM677 | 4 | 85,785 | 35,621 | |
| 60 days | Control | 1 | 59,970 | 24,542 | |
|  | Control | 2 | 62,488 | 26,763 | |
|  | Control | 3 | 22,255 | 8,148 | |
|  | LBUM677 | 1 | 31,777 | 11,562 | |
|  | LBUM677 | 2 | 55,322 | 21,965 | |
|  | LBUM677 | 4 | 55,406 | 21,858 | |
| 90 days | Control | 1 | 55,825 | 22,711 | |
|  | Control | 2 | 78,206 | 35,418 | |
|  | Control | 3 | 64,021 | 28,115 | |
|  | Control | 4 | 49,481 | 20,319 | |
|  | LBUM677 | 1 | 54,017 | 21,410 | |
|  | LBUM677 | 2 | 20,676 | 7,804 | |
|  | LBUM677 | 3 | 36,580 | 12,998 | |
